# Baihe Zhimu Decoction ameliorates anxious depression via PI3K/AKT/mTOR pathway‐mediated neuroprotection and inhibition of hippocampal neuronal apoptosis

**DOI:** 10.1002/ame2.70295

**Published:** 2026-10-12

**Authors:** Yuanshan Han, Kexin He, Haolan Yang, Jiacheng Dai, Ronglin Chen, Yang Liu, Yuhong Wang, Lin Tang, Hongqing Zhao

**Affiliations:** ^1^ Hunan Key Laboratory of Traditional Chinese Medicine Prevention & Treatment of Depressive Diseases Hunan University of Chinese Medicine Changsha Hunan China; ^2^ The First Affiliated Hospital Hunan University of Chinese Medicine Changsha Hunan China; ^3^ School of Medicine Hunan University of Chinese Medicine Changsha Hunan China; ^4^ Academy of Chinese Medical Sciences/Science & Technology Innovation Center Hunan University of Chinese Medicine Changsha Hunan China

**Keywords:** anxious depression, Baihe Zhimu Decoction, network pharmacology, neuronal apoptosis, PI3K/AKT/mTOR signaling pathway

## Abstract

**Background:**

Anxious depression involves severe emotional dysregulation with a high risk of relapse and suicide. Although the classical Traditional Chinese Medicine formula Baihe Zhimu Decoction (BZD) is widely used for affective disorders, its underlying mechanisms remain poorly understood.

**Methods:**

In this study, the chemical constituents of BZD were first identified using UPLC‐Q‐TOF‐MS/MS, followed by a maximum‐dose safety evaluation. The therapeutic effects of BZD were then investigated in an anxious‐depression mouse model induced by chronic restraint stress (CRS) combined with corticosterone (CORT) administration. Behavioral assessments, including the open field test (OFT), elevated plus maze (EPM), sucrose preference test (SPT), and forced swim test (FST), were performed, together with histopathological examination of hippocampal tissues. Network pharmacology was employed to analyze the relationships between active compounds and potential targets and to predict related signaling pathways. Subsequently, western blot analysis, immunofluorescence staining, Hoechst staining, and flow cytometry, combined with pathway‐specific inhibitors, were used to examine the involvement of the signaling pathway in hippocampal tissues and primary hippocampal neurons.

**Results:**

Forty‐one constituents were identified in BZD. In vivo results showed that BZD significantly alleviated hippocampal pathological injury and improved anxiety‐ and depression‐like behaviors in stressed mice. Network pharmacology analysis identified 19 active compounds and 622 potential targets, highlighting the PI3K/AKT/mTOR signaling pathway. Mechanistically, BZD activated this pathway and protected against neuronal injury, while these effects were attenuated by the PI3K inhibitor LY294002.

**Conclusions:**

BZD exerts potent neuroprotective and therapeutic effects against anxious depression, primarily driven by activating the PI3K/AKT/mTOR pathway and preserving hippocampal neuronal integrity.

## Graphical abstract

Baihe Zhimu Decoction alleviates anxious depression‐like behaviors and protects hippocampal neurons. Chemical profiling and network pharmacology reveal multiple active components acting via the PI3K/AKT/mTOR pathway, enhancing neuronal survival and function, as confirmed by multi‐level in vitro and in vivo validation.

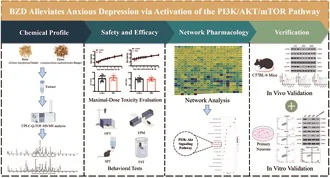

## 1 INTRODUCTION

Anxious depression is characterized by significant anxiety symptoms accompanying depressive episodes. Approximately 50%–75% of patients with major depressive disorder (MDD) meet DSM‐5 criteria for “with anxious distress”.^1^ Compared with non‐anxious depression, anxious depression is associated with a more severe clinical course, higher suicide risk, poor antidepressant response, and increased comorbidity with other medical conditions.^2^, ^3^ Current treatments rely primarily on selective serotonin reuptake inhibitors (SSRIs), serotonin‐norepinephrine reuptake inhibitors (SNRIs), and cognitive‐behavioral therapy; however, patients with anxious depression generally show lower response rates, unstable treatment efficacy, and experience side effects such as nausea and vomiting.^4^, ^5^ Hence, there is a critical clinical demand for the development of more selective and safer treatment modalities with fewer adverse reactions.

Baihe Zhimu Decoction (BZD), originally described in Jin Gui Yao Lue by Zhang Zhongjing during the Eastern Han dynasty, consists of fresh lily bulbs and dried *Anemarrhena asphodeloides* rhizomes. It has the functions of nourishing yin, clearing heat, and relieving irritability. Clinically, BZD is used to treat “lily disease,” which manifests as restlessness, insomnia, low mood, and anxiety—symptoms analogous to modern anxious depression. Previous studies have shown that BZD is widely used for depression, anxiety, insomnia, and postpartum depression, with good tolerability and fewer side effects compared with some traditional medications.^6^ Pharmacological studies suggest that its mechanisms may involve neuroendocrine regulation,^7^ neurotransmitter modulation,^8^ and anti‐inflammatory activities.^9^ However, most existing research focuses on chemical composition analysis^8^ or network‐pharmacology‐based target prediction,^10^ and systematic experimental verification of BZD's molecular mechanism is lacking.

Network pharmacology, a systems‐biology‐based method, integrates multiple databases to construct drug‐component‐target networks, enabling prediction of key active components, targets, and pathways in multi‐component herbal formulas.^11^ Although this approach aligns well with the holistic nature of traditional Chinese medicine (TCM),^12^ it also has limitations—most studies rely on predicted oral bioavailability or drug‐likeness, and neglect actual exposure levels and metabolites.^13^ Therefore, combining network pharmacology with experimental validation is essential to enhance credibility.

Using UPLC‐Q‐TOF‐MS/MS, the chemical constituents of BZD were systematically analyzed and its safety profile was evaluated. BZD was then administered to an anxious‐depression mouse model induced by chronic restraint stress (CRS) combined with corticosterone (CORT) administration to assess its anxiolytic and antidepressant effects. Based on the identified chemical components, network pharmacology was employed to predict the potential pathways and targets through which BZD may exert therapeutic effects on anxiety‐depression. Finally, comprehensive in vivo and in vitro experiments were conducted to validate these predictions, with a particular focus on the PI3K/AKT/mTOR signaling pathway, a key regulator of neuronal survival and neuroprotection. This study aims to elucidate the neuroprotective mechanisms of BZD in anxious depression, thereby providing a scientific basis for its mode of action and clinical application.

## 2 METHODS

### 2.1 Animals

A total of 40 ICR mice (18–22 g; equal numbers of males and females) were used for the acute toxicity study, based on their high genetic diversity and widespread use in toxicological assessments.^14^ In contrast, 60 male C57BL/6 mice (18–22 g) were used for the subsequent efficacy and mechanistic experiments, as this inbred strain has well‐characterized behavioral and neurobiological profiles, which ensure reproducibility and sensitivity in modeling anxiety‐ and depression‐like behaviors.^15^ All animals were purchased from Hunan Slake Jingda Laboratory Animal Co., Ltd. [SCXK (Xiang) 2021‐0002]. All mice were housed in the Experimental Animal Center of Hunan University of Chinese Medicine [SYXK (Xiang) 2019‐0009] under controlled temperature (23 ± 2°C) and humidity (55% ± 5%), with free access to food and water. All animal procedures were reviewed and approved by the Animal Ethics Committee of Hunan University of Chinese Medicine (Ethics approval No. LLBH‐202308120003).

### 2.2 Preparation of BZD

BZD was prepared from Baihe *(Lilium lancifolium* Thunb. 30 g) and Zhimu (*Anemarrhena asphodeloides* Bunge, 10 g), according to the prescription in the 2020 Chinese Pharmacopeia. The two herbs were decocted separately twice with water (10× and 6×, w/v), and the filtrates were combined. The extracts were mixed, concentrated, and adjusted to 1 g/mL, calculated based on the weight of the crude herbs.

### 2.3 Chemical component analysis of BZD

A 1 g/mL BZD decoction was mixed with methanol (1:20, v/v), ultrasonicated for 20 min, centrifuged at 10 000 rpm for 5 min, and filtered (0.22 μm) prior to analysis. Mangiferin (batch no. 111607‐201704) and Timosaponin BII (batch no. 111839‐202107) were obtained from the National Institutes for Food and Drug Control (China). Timosaponin AIII (CAS: 41059‐79‐4), Neomangiferin (CAS: 64809‐67‐2), Isomangiferin (CAS: 24699‐16‐9), Regaloside B (CAS: 114420‐67‐6), and Timosaponin BIII (CAS: 142759‐74‐8) were purchased from Chengdu Pufeide Biotechnology Co., Ltd. Standard solutions were prepared at 0.2 mg/mL in methanol, then mixed and filtered for analysis.

Chromatographic separation was achieved on an Agilent ZORBAX Eclipse Plus C18 column (3.0 × 100 mm, 1.8 μm) using a mobile phase of acetonitrile (A) and water containing 0.05 mmol/L ammonium formate (B). The gradient elution was 5%–15% A (0–5 min), 15%–35% A (5–10 min), 35%–65% A (10–20 min), and 65%–95% A (20–30 min) at 0.4 mL/min (1 μL injection). MS analysis was performed on an Agilent 1290 UPLC‐6540 Q‐TOF in both ESI^+/−^ modes (MS1 *m/z* 100–1700, MS2 *m/z* 50–1000). Source parameters included: drying gas (325°C, 6.8 L/min), sheath gas (350°C), capillary voltage (4.0 kV), and fragmentor voltage (150 V). Data were processed via MassHunter B.07.00.

### 2.4 Maximal‐dose toxicity evaluation of BZD

Acute oral toxicity of BZD was evaluated in ICR mice. Mice (18–22 g) were divided into control and BZD groups (*n* = 10/sex/group). After 12 h fasting, BZD was administered by gavage at a concentration of 3.2653 g/mL crude drug and a volume of 40 mL/kg, divided into two doses within 24 h; controls received equivalent volumes of distilled water. Animals were observed for 24 h and monitored for 14 days for clinical signs, mortality, and body weight. Blood and liver samples were collected for hematology, liver function, and H&E histopathology.

### 2.5 Model establishment and drug administration

Venlafaxine hydrochloride tablets (75 mg/tablet, batch No. 220819) were obtained from Chengdu Kanghong Pharmaceutical Group Co., Ltd. Sixty male C57BL/6 mice^16^ were randomly assigned to six groups: control, model, venlafaxine (Ven), and BZD high‐, medium‐, and low‐dose groups (BZD‐H, BZD‐M, BZD‐L). After one week of acclimatization, all groups except the control were subjected to chronic restraint stress (6 h/day) combined with daily subcutaneous CORT (30 mg/kg) injection for 28 days to induce an anxious depression model. From week 2 to week 4, mice received corresponding treatments. BZD was administered by oral gavage at 2.6, 5.2, and 10.4 g/kg. The medium dose (5.2 g/kg) was calculated from the clinical daily dose using the body surface area normalization method, while the low‐ and high‐dose groups were set at 0.5‐ and 2‐fold of the equivalent dose, respectively.^17^ The Ven group received venlafaxine (20 mg/kg),^18^ while control and model groups were given saline. Behavioral tests were conducted after the treatment period. The high‐dose BZD group (10.4 g/kg), which exhibited the greatest therapeutic efficacy, was selected for Western blot analysis.

### 2.6 Behavioral tests

Behavioral tests were conducted in the following order: the Open Field Test (OFT), Elevated Plus Maze (EPM), Sucrose Preference Test (SPT), and Forced Swim Test (FST). This sequence was selected in accordance with common practice, progressing from relatively less stressful to more stressful behavioral paradigms, thereby minimizing potential carryover effects and the cumulative impact of stress on subsequent assessments.^19^

#### 2.6.1 Open Field Test (OFT)

The OFT assesses locomotor activity, exploration, and anxiety in a novel environment, with reduced distance traveled in the center indicating higher anxiety. The OFT was performed in a 40 cm × 40 cm × 40 cm arena. Mice were placed in the center and allowed to explore freely for 5 min. Total distance traveled and distance traveled in the central area were recorded using a behavioral tracking system. The apparatus was cleaned with 75% ethanol between trials.

#### 2.6.2 Elevated Plus Maze (EPM)

The EPM is a widely used test for assessing anxiety‐like behavior, based on the conflict between exploration of a novel environment and fear of open elevated arms. The EPM test was conducted to assess anxiety‐like behavior. Mice were placed at the center of the maze facing an open arm and observed for 5 min. The number of entries and time spent in open and closed arms were recorded, and open‐arm entry (OE%) and time (OT%) were calculated.

#### 2.6.3 Sucrose Preference Test (SPT)

The SPT was used to evaluate anhedonia, a core symptom of depression. Mice were habituated to 2% sucrose solution for 24 h, followed by 24 h with access to both sucrose solution and water. After 12 h of water deprivation, a 2 h test was conducted. Sucrose preference (%) was calculated as sucrose intake divided by total fluid intake.

#### 2.6.4 Forced Swim Test (FST)

The FST was used to assess behavioral despair in rodents. Mice were placed in water (22–24°C). After 1 min of adaptation, immobility time during the subsequent 5 min was recorded.

### 2.7 Histopathological staining

After euthanasia, hippocampal tissues were rapidly dissected and fixed in 4% paraformaldehyde or electron microscopy fixative. For histopathological analysis, tissues were dehydrated, paraffin‐embedded (BM‐VIII, Hongye, China), and sectioned at 5 μm using a microtome (RM223, Leica, Germany), followed by hematoxylin–eosin and Nissl staining for morphological observation.

### 2.8 Transmission electron microscopy (TEM)

For transmission electron microscopy, tissues were fixed at 4°C for 24 h, dehydrated, resin‐embedded, and ultrathin sections (~50 nm) were prepared. Sections were stained with uranyl acetate and lead citrate and examined under a transmission electron microscope (HT7700, Hitachi, Japan) to assess neuronal ultrastructure.

### 2.9 Network pharmacology analysis

The chemical constituents of BZD, previously identified by UPLC‐Q‐TOF‐MS/MS analysis, were screened using SwissADME,^20^ and compounds with high gastrointestinal absorption and drug‐likeness ≥3 were retained. Drug‐likeness was assessed based on five established drug‐likeness filters, namely Lipinski, Ghose, Veber, Egan, and Muegge. A compound was considered to satisfy the drug‐likeness criterion when it fulfilled at least three of these five rules. Potential targets were predicted using SwissTargetPrediction^21^ (http://www.swisstargetprediction.ch/), and targets with non‐zero probability scores were retained for subsequent network pharmacology analyses based on previously reported approaches.^22^, ^23^

Anxious depression‐related targets were obtained from GeneCards^24^ (https://www.genecards.org/) using “anxious depression” as the keyword. A protein–protein interaction (PPI) network was constructed using STRING, and key targets were identified using CytoHubba based on MCC and Degree values.

Overlapping targets between BZD and disease were used to construct a component‐target network in Cytoscape and subjected to GO and KEGG enrichment analyses using the R package “clusterProfiler”.

### 2.10 Primary neuron culture

Primary hippocampal neurons were isolated from embryonic day 16–18 Sprague–Dawley (SD) rats. SD rat primary neurons are characterized by good stability, high cell yield and simple culture procedures, and have been widely recognized as a classic in vitro model for exploring neuronal molecular mechanisms.^25^, ^26^ Hippocampi were dissected and enzymatically digested with trypsin and collagenase, and cells were plated onto poly‐L‐lysine‐coated plates. Cells were cultured in Neurobasal medium supplemented with B27 and Glutamax (Thermo Fisher Scientific, USA) and maintained at 37°C with 5% CO_2_ in an incubator (ESCO, Singapore). Half of the medium was replaced every 3 days, and neurons were used for experiments at 7–8 days in vitro. Cellular morphology was observed under an inverted microscope (Zeiss, Germany), and neuronal purity was confirmed by NSE immunofluorescence.

### 2.11 Preparation of drug‐containing serum

SD rats were randomly divided into three groups: control, BZD, and Ven groups. Rats in the control group received an equivalent volume of distilled water by oral gavage, whereas rats in the BZD group were administered BZD at a high dose of 10.4 g/kg. Rats in the Ven group received venlafaxine at a dose of 20 mg/kg. All treatments were administered twice daily for 7 consecutive days. Before the final administration, the rats were fasted for 12 h with free access to water. One hour after the final gavage, the rats were anesthetized, and blood samples were collected from the abdominal aorta. The blood samples were allowed to clot and then centrifuged to separate the serum. The collected serum was heat‐inactivated at 56°C for 30 min, filtered through a 0.22 μm microporous membrane, aliquoted, and stored at −80°C until further use.

### 2.12 Cell grouping, modeling, and drug treatment

To determine the optimal concentration of BZD‐medicated serum, primary neurons were treated with 5%, 10%, 15%, and 20% (v/v) serum, and cell viability was assessed by MTT assay. For the CORT‐induced injury model, neurons were exposed to 200 μmol/L CORT for 24 h and treated with different concentrations of BZD‐medicated serum or a positive control.^27^ Cell viability was measured to evaluate neuroprotective effects. To assess neuronal morphology and function, neurons were treated with 10% BZD‐medicated serum, and NeuN immunofluorescence staining and neurotransmitter assays were performed. To investigate the involvement of the PI3K/AKT/mTOR pathway, neurons were treated with BZD in the presence or absence of the PI3K inhibitor LY294002. Apoptosis was analyzed by Hoechst staining and flow cytometry, and the expression of apoptosis‐related and pathway‐related proteins was evaluated by RT‐qPCR and Western blot.

### 2.13 Immunofluorescence staining

Neurons were fixed (4% PFA, 15 min), permeabilized (0.3% Triton X‐100, 10 min), and blocked (goat serum, 30 min). The neurons were incubated with primary antibodies against NSE (1:300, 66 150‐1‐Ig, Proteintech) and NeuN (1:300, 26 975‐1‐AP, Proteintech) overnight at 4°C. Secondary antibodies (Cy3‐anti‐rabbit, E‐AB‐1010; Alexa Fluor 488‐anti‐mouse, E‐AB‐1056; Elabscience) were applied at 37°C for 1 h. After DAPI staining, images were captured using a Zeiss LSM 900 confocal microscope.

### 2.14 MTT assay

Hippocampal neurons (1 × 10^5^ cells/mL) were seeded in 96‐well plates (*n* = 6). Post‐treatment, medium was replaced with 0.5 mg/mL MTT (Sigma, M2003). After a 4‐h incubation, formazan was dissolved in 150 μL DMSO by shaking for 10 min. Absorbance at 490 nm was measured. Viability was calculated as:

$$\text{Cell viability}\;\left(\%\right)=\left[\left(A_{\text{treatment}}–A_{\text{blank}}\right)/\left(A_{\text{control}}–A_{\text{blank}}\right)\right]\times 100\%$$

### 2.15 Hoechst 33342 staining

Hippocampal neurons (1 × 10^5^ cells/mL) in 96‐well plates were washed with PBS post‐treatment. Cells were incubated with 5 μg/mL Hoechst 33342 in the dark for 10 min. After 2–3 PBS washes to remove excess dye, nuclear morphology was captured via fluorescence microscopy.

### 2.16 Flow cytometry

Post‐treatment, hippocampal neurons from 6‐well plates were resuspended in 100 μL 1× Binding Buffer (1 × 10^6^ cells/mL). Cells were labeled with 5 μL Annexin V‐FITC and 5 μL PI (E‐CK‐A211, Elabscience) for 15 min at room temperature in the dark. After adding 400 μL Binding Buffer, apoptosis was immediately quantified via flow cytometry.

### 2.17 Enzyme‐linked immunosorbent assay (ELISA)

ELISA kits were used according to manufacturer's instructions to measure 5‐hydroxytryptamine (5‐HT), dopamine (DA), glutamate (Glu) and γ‐aminobutyric acid (GABA) levels in cell culture supernatants.

### 2.18 RT‐qPCR

RT‐qPCR Total RNA was extracted from hippocampal tissue (~100 mg) using the TRIzol method. RNA purity (A_260_/A_280_ ratio ≈ 2.0) and concentration were verified via NanoDrop. Equal RNA amounts were reverse‐transcribed to cDNA per kit instructions. qPCR was performed on a K960 system (Hangzhou Jinge, China) with the following thermal profile: 95°C for 15 min, then 40 cycles of 95°C (10 s) and 60°C (30 s). Target gene Cq values were normalized to β‐actin. Primers are listed in Table S1.

### 2.19 Western blot

Tissues and cells were lysed in RIPA buffer (1% PMSF) and centrifuged (12 000 × g, 15 min, 4°C). Proteins (30 μg) were resolved via SDS‐PAGE, transferred to PVDF membranes, and blocked with 5% milk (1.5 h). Membranes were incubated overnight at 4°C with primary antibodies (details in Table S2), followed by 1‐h incubation with HRP‐conjugated secondary antibodies (1:15 000). Signals were captured using ChemiDoc XRS+ (Bio‐Rad) and quantified via ImageJ. Target levels were normalized to β‐actin.

### 2.20 Statistical analysis

GraphPad Prism 9.0 software was used for statistical analyses and graphing. Data are presented as mean ± standard deviation (*x̄* ± *s*). One‐way analysis of variance (ANOVA) was used for multiple‐group comparisons, and a LSD‐*t* test was used for pairwise comparisons. *p* < 0.05 was considered statistically significant.

## 3 RESULTS

### 3.1 Chemical constituents of BZD

A total of 41 chemical constituents were identified in BZD by mass spectrometry, mainly including phenylpropanoids, flavonoids, and saponins. Phenylpropanoids were represented by regalosides, flavonoids by mangiferin, isomangiferin, neomangiferin and their derivatives, and saponins by timosaponins. Small amounts of sugars, amino acids, and other compounds were also detected. Detailed information is shown in Table S3, and base peak ion chromatograms are presented in Figure 1A,B. Representative compounds were used to prepare mixed standards for verification. Their chromatographic peaks (Figure 1C) showed consistent retention times and fragment ions with those in BZD, confirming the identification of major constituents.

**FIGURE 1:**
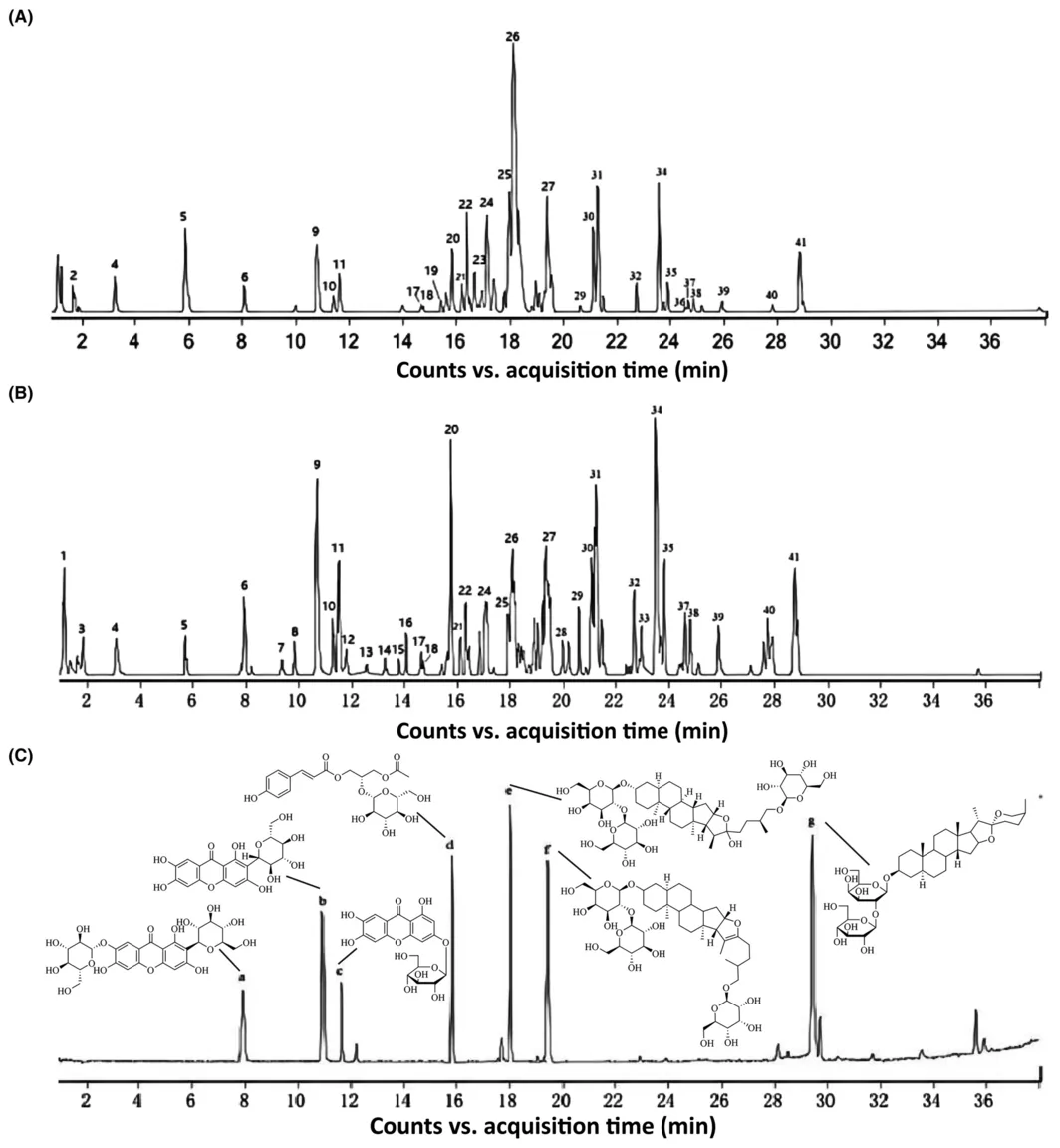
UPLC‐Q‐TOF‐MS/MS analysis of BZD. (A) Total ion chromatogram (TIC) of BZD in positive ion mode. (B) TIC of BZD in negative ion mode. The numbers correspond to the peak numbers of the 41 identified compounds listed in Table S3. (C) Chromatogram of a mixed standard solution containing seven reference compounds and their corresponding chemical structures: a, neomangiferin; b, mangiferin; c, isomangiferin; d, timosaponin II; e, timosaponin B; f, timosaponin BIII; g, timosaponin AIII. These standards were used to verify the retention times and fragmentation characteristics of the corresponding compounds in the BZD sample.

### 3.2 Acute toxicity evaluation of BZD at the maximum administration dose

The maximum dose of BZD was 261.224 g/kg, equivalent to 50.23‐fold (body surface area) and 457.14‐fold (body weight) of the clinical dose. No deaths occurred within 14 days. Body weight decreased on day 1 (*p* < 0.01) but recovered from day 2 with no difference vs. controls (*p* > 0.05) (Figure 2A,B). Hematological parameters showed no significant changes (*p* > 0.05) (Table 1), and AST and ALT levels were unchanged (Figure 2C,D). Histology showed normal liver architecture without inflammation or necrosis (Figure 2E). These results indicate no acute toxicity or liver injury at this dose.

**FIGURE 2:**
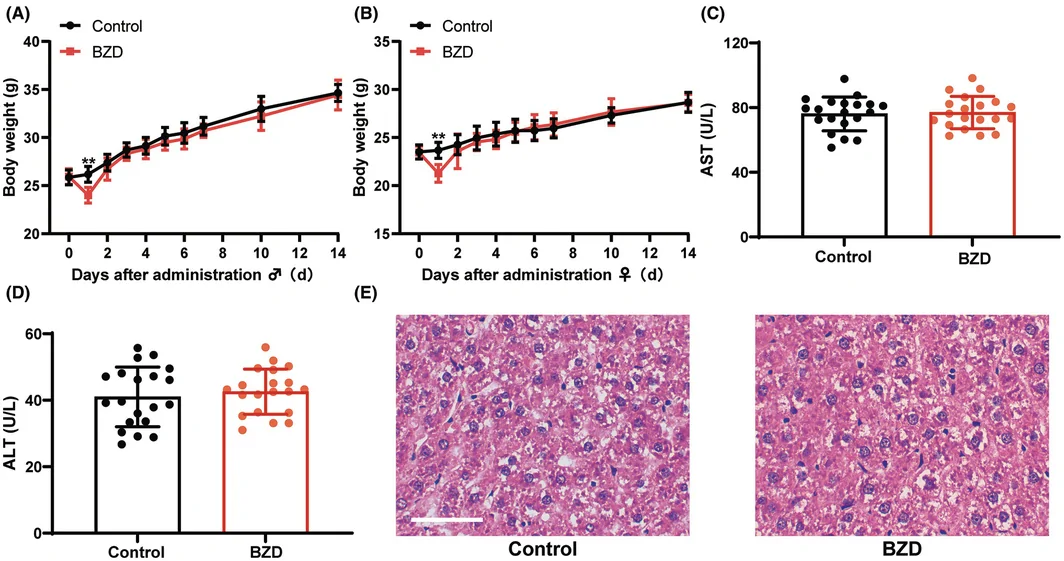
Acute oral toxicity evaluation of BZD at the maximum dose. (A, B) Body weight changes in male and female mice within 14 days after administration (*n* = 10). (C, D) Serum AST (aspartate aminotransferase) and ALT (alanine aminotransferase) levels (*n* = 20). (E) Representative H&E staining of liver tissue (×200), scale bar: 100 μm. No significant histopathological abnormalities were observed. Data are presented as mean ± SD. **p < 0.01 *vs*. control group.

**TABLE 1:** Comparison of hematological parameters between BZD‐treated group and distilled water control group.

Group	Dosage (g/kg)	WBC(10^9^/L)	RBC(10^12^/L)	HGB(g/L)	PLT(10^9^/L)	MCH(pg)	MCV(fL)	MPV(fL)
Control	/	5.40 ± 1.38	6.71 ± 0.71	110.85 ± 8.44	724.05 ± 144.08	16.47 ± 0.86	52.34 ± 1.42	8.00 ± 0.53
BZD	130.612	5.46 ± 1.56	7.05 ± 0.69	115.50 ± 9.75	708.75 ± 126.97	16.24 ± 0.83	51.84 ± 2.65	8.15 ± 0.57

*Note*: Data were analyzed using Student's *t*‐test and presented as mean ± standard deviation (*n* = 20) (*p* > 0.05).

Abbreviations: BZD, Baihe Zhimu Decoction; HGB, hemoglobin; MCH, mean corpuscular hemoglobin; MCV, mean corpuscular volume; MPV, mean platelet volume; PLT, platelet count; RBC, red blood cell count; WBC, white blood cell count.

### 3.3 BZD effectively reversed CRS + CORT‐induced anxiety‐ and depression‐like behaviors

The experimental flowchart is shown in Figure 3A. Anxiety‐like and depression‐like behaviors were assessed by OFT EPM and SPT FST, respectively. Compared with the normal group, the model group showed reduced total distance and central distance in the OFT, decreased OT% and OE% in the EPM (*p* < 0.01), lower sucrose preference, and longer immobility time in the FST, indicating anxiety‐ and depression‐like behaviors (Figure 3B–I). Compared with the model group, BZD significantly improved these behavioral abnormalities at all doses (*p* < 0.05 or *p* < 0.01). Venlafaxine showed similar effects. These results indicate that BZD ameliorated CRS combined with CORT‐induced anxiety‐ and depression‐like behaviors in mice.

**FIGURE 3:**
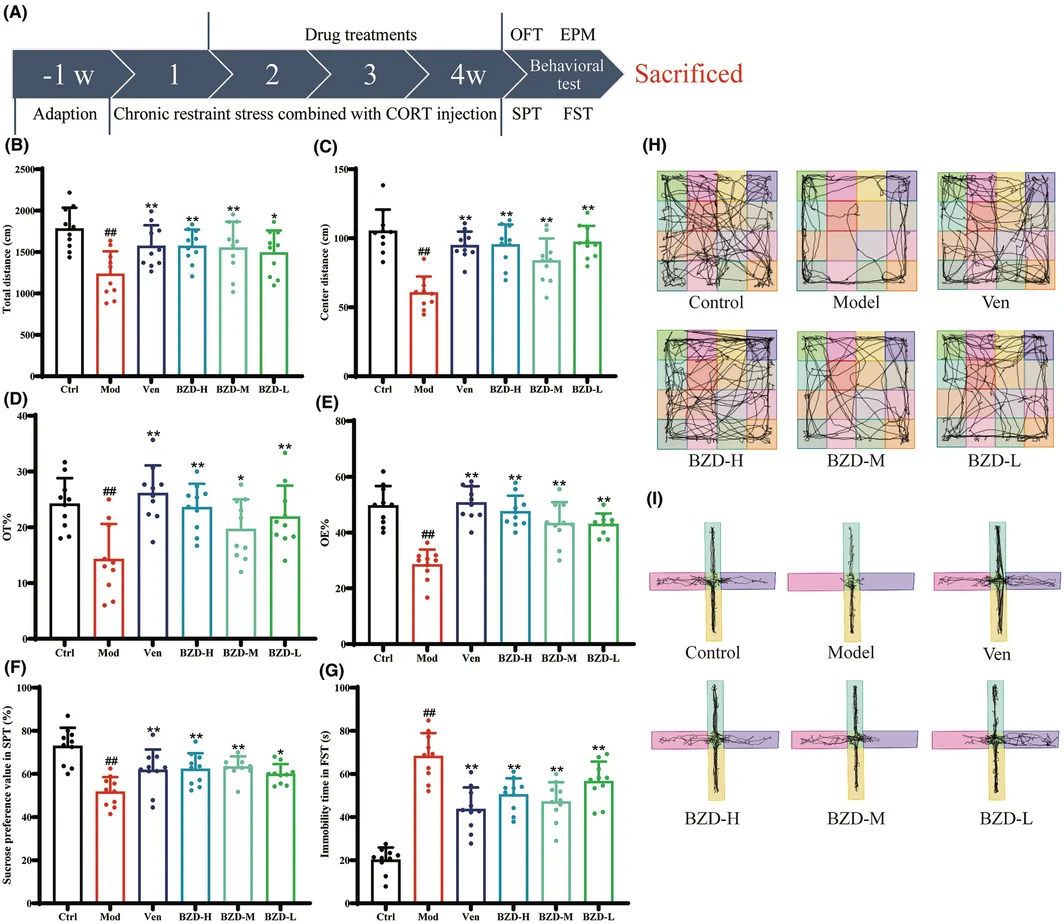
Effects of BZD on CRS combined with CORT‐induced anxiety‐ and depression‐like behaviors in mice. (A) Experimental flowchart. (B, C) Total distance traveled and distance traveled in the center area in the OFT. (D, E) Percentage of open‐arm entries (OE%) and percentage of time spent in open arms (OT%) in the EPM. (F) Sucrose preference in the SPT. (G) Immobility time in the FST. (H) Representative OFT movement trajectories. (I) Representative EPM movement trajectories. Data are presented as mean ± SD (*n* = 10). ^##^ *p* < 0.01 *vs*. control group; *p < 0.05, **p < 0.01 *vs*. model group.

### 3.4 BZD exerts protective effects on hippocampal neurons and synapses

The hippocampus is a key region of the central nervous system involved in emotional regulation and memory consolidation, contributing to complex neural processes such as behavior, learning, and emotional responses.^28^ H&E, Nissl staining, and TEM were performed to assess neuronal morphology and synaptic integrity in the hippocampal CA1 region. In the model group, neurons were reduced in number, disorganized, and exhibited shrunken morphology (Figure 4A), accompanied by decreased Nissl bodies (Figure 4B) and ultrastructural damage, including mitochondrial swelling, disrupted cristae, and reduced synaptic density (Figure 4C). BZD and venlafaxine markedly alleviated these changes, preserving neuronal structure and improving synaptic organization, with increased Nissl bodies. These results indicate that BZD ameliorates neuronal and synaptic damage in the hippocampus.

**FIGURE 4:**
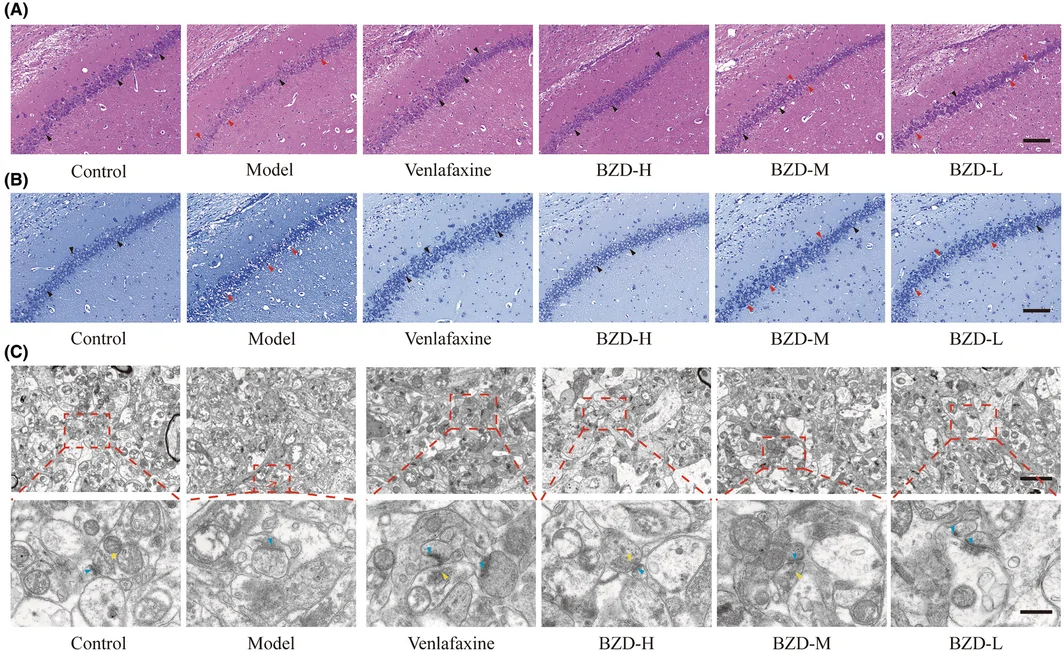
Effects of BZD on neuronal and synaptic structure in the hippocampal CA1 region. (A) H&E staining of the hippocampal CA1 region (×200), scale bar: 100 μm. Black arrows indicate normal neurons, whereas red arrows indicate damaged neurons. (B) Nissl staining of the hippocampal CA1 region (×200), scale bar: 100 μm. Black arrows indicate normal neurons, whereas red arrows indicate damaged neurons. (C) Representative TEM images of hippocampal synaptic ultrastructure (×5000, ×20 000), scale bars: 2 μm and 500 nm. Blue arrows indicate the synaptic cleft, and yellow arrows indicate synaptic vesicles.

### 3.5 Network pharmacology prediction of the anti‐anxiety and antidepressant mechanisms of BZD

Based on the chemical constituents identified by UPLC‐Q‐TOF‐MS/MS, network pharmacology screened 19 active compounds from Baihe and Zhimu. A total of 622 anxiety‐ and depression‐related targets were obtained from SwissTargetPrediction and GeneCards. The PPI network (Figure 5A) and the compound–target–drug network (Figure 5B) were constructed, and CytoHubba analysis identified 174 key targets based on MCC and Degree values, among which AKT1, PIK3CA, MTOR, and EGFR were central. GO and KEGG analyses were performed on intersecting targets. GO results showed associations with neural activity and emotional regulation, including synaptic membrane, dendritic spine, and neurotransmitter receptor activity (Figure 5C), suggesting involvement in synaptic transmission and neuroplasticity. KEGG enrichment analysis identified multiple pathways associated with emotional disorders. Among them, the PI3K‐Akt signaling pathway was significantly enriched (Count = 9, adjusted *p*‐value = 0.0012) and involved several key targets, including AKT1, PIK3CA, and MTOR (Figure 5D). Given the central positions of these targets in the PPI network and the established role of the PI3K/AKT/mTOR pathway in neuronal survival and emotional regulation, this pathway was selected for subsequent experimental validation.

**FIGURE 5:**
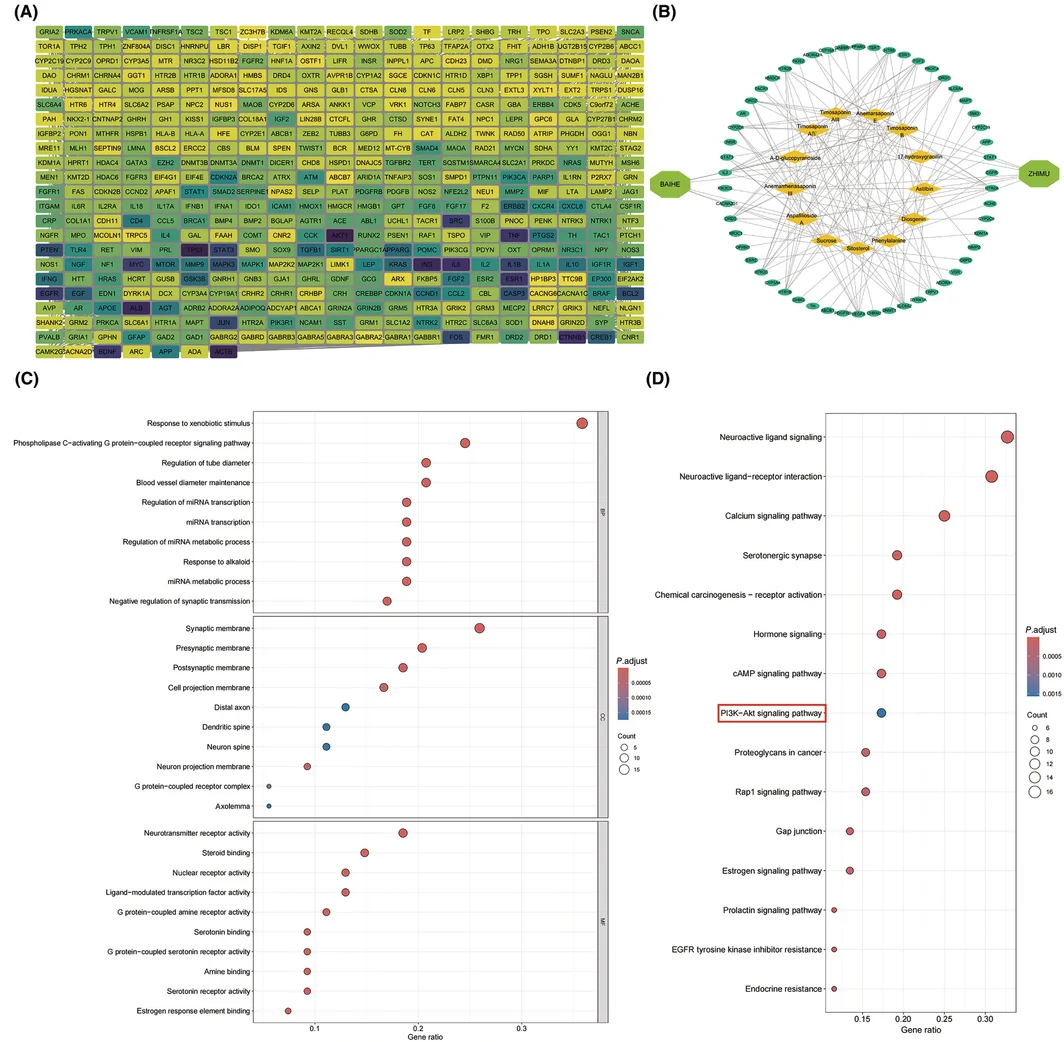
Network pharmacology analysis of BZD in the treatment of anxious depression. (A) Core protein–protein interaction (PPI) network of BZD and anxious depression. The color intensity of nodes reflects Degree values. (B) “Herb‐compound‐target” network of BZD. (C) GO enrichment analysis of potential targets of BZD active components. (D) KEGG pathway enrichment analysis of potential targets. The red box highlights the PI3K‐Akt signaling pathway (Count = 9, adjusted *p*‐value = 0.0012).

### 3.6 BZD activates the PI3K/AKT/mTOR pathway in vivo and inhibits hippocampal neuronal apoptosis

To validate the mechanism predicted by network pharmacology, the PI3K/AKT/mTOR pathway and apoptosis‐related molecules were examined. Compared with the normal group, AKT and mTOR mRNA levels and the phosphorylation of PI3K, AKT, and mTOR were significantly decreased in the model group (Figure 6A,B). BZD treatment markedly restored these changes. In addition, the protein expression of Bcl‐2 was downregulated, whereas Bax and cleaved‐caspase‐3 were upregulated in the model group; BZD reversed these alterations (Figure 6C). RT‐qPCR results showed similar trends (Figure 6A). These findings indicate that BZD alleviates neuronal apoptosis by activating the PI3K/AKT/mTOR pathway. The corresponding full‐length Western blot images are provided in Figure S1.

**FIGURE 6:**
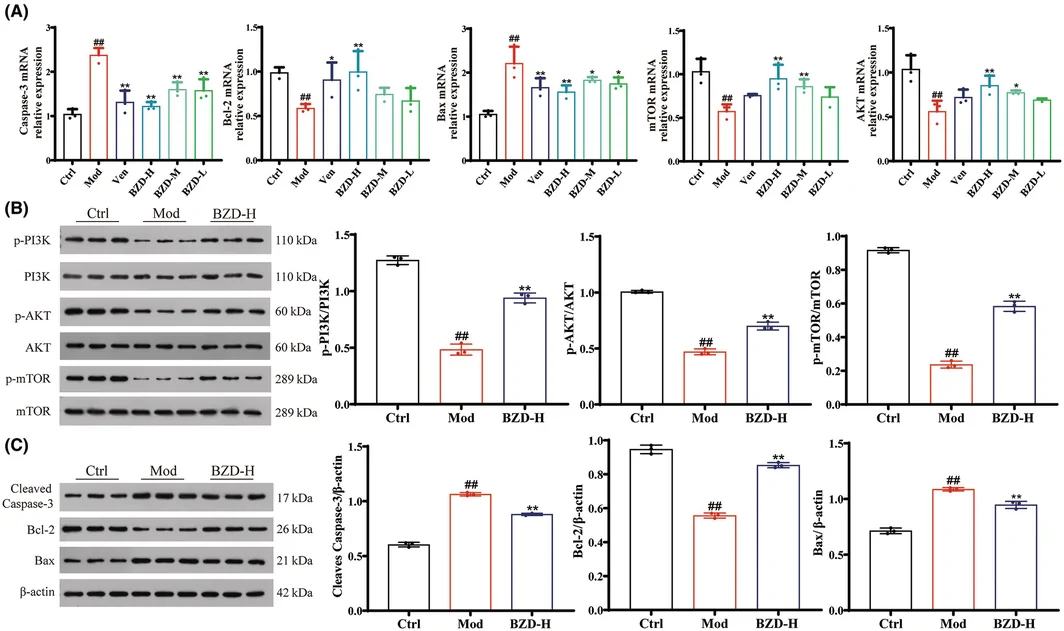
In vivo effects of BZD on the PI3K/AKT/mTOR pathway. (A) Relative mRNA expression levels of AKT, mTOR, and apoptosis‐related genes determined by RT‐qPCR. (B) Western blot analysis of p‐PI3K, PI3K, p‐AKT, AKT, p‐mTOR, and mTOR protein levels in hippocampal tissue. (C) Western blot analysis of apoptosis‐related proteins in hippocampal tissue. Data are presented as mean ± SD (*n* = 3). ^##^ *p* < 0.01 *vs*. control group; *p < 0.05, **p < 0.01 *vs*. model group.

### 3.7 BZD‐containing serum ameliorates CORT‐induced damage in primary hippocampal neurons

NSE immunofluorescence confirmed the neuronal identity of the primary hippocampal cultures. The majority of DAPI‐positive cells were also positive for NSE, indicating a high purity of the neuronal cultures (Figure 7A). The cytotoxicity and protective effects of BZD‐containing serum were then evaluated (Figure 7B,C), showing that 20% serum exhibited cytotoxicity, whereas 10% serum provided optimal protection against CORT‐induced injury and was used for subsequent experiments. NeuN staining and neurotransmitter analysis (Figure 7D,E) revealed that CORT reduced NeuN‐positive cells and levels of 5‐HT, DA, and GABA, while increasing glutamate (Figure 7F–I). Treatment with 10% BZD‐containing serum or venlafaxine significantly reversed these changes. These results indicate that BZD‐containing serum alleviates CORT‐induced neuronal injury and restores neurochemical balance.

**FIGURE 7:**
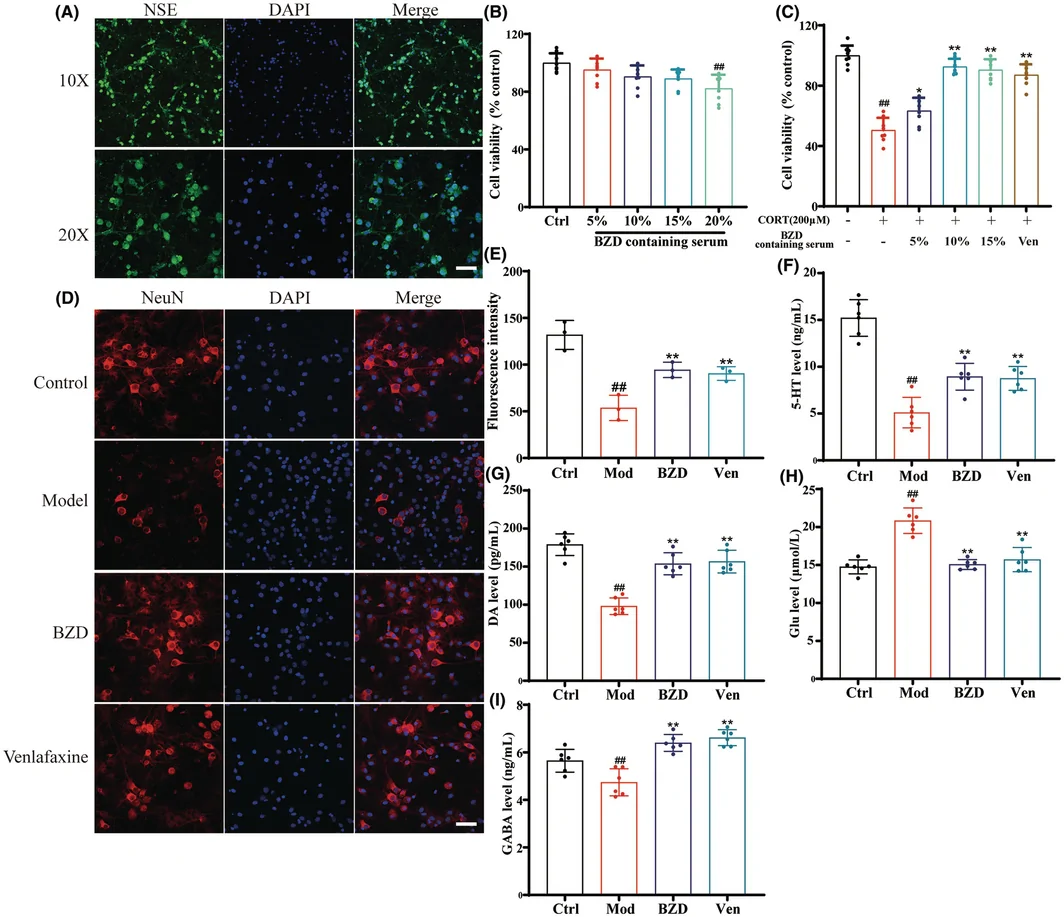
Protective effects of BZD‐containing serum against CORT‐induced injury in primary hippocampal neurons. (A) Immunofluorescent co‐localization of NSE (green) and DAPI (blue), scale bar: 50 μm. (B) Cell viability after treatment with different concentrations of BZD‐containing serum for 24 h (MTT assay, *n* = 6). (C) Protective effects of different concentrations of BZD‐containing serum against CORT‐induced cytotoxicity (*n* = 6). (D, E) NeuN staining and quantitative fluorescence analysis. (F–I) Levels of 5‐HT, DA, Glu, and GABA in cell supernatants (*n* = 8). Data are presented as mean ± SD. ^##^ *p* < 0.01 *vs*. control group; *p < 0.05, **p < 0.01 *vs*. model group.

### 3.8 BZD‐containing serum inhibits hippocampal neuron apoptosis by activating the PI3K/AKT/mTOR pathway in vitro

The effects of BZD‐containing serum on CORT‐induced neuronal apoptosis and the involvement of the PI3K/AKT/mTOR pathway were further examined using LY294002. CORT markedly induced apoptosis, as shown by nuclear condensation (Figure 8A), increased apoptosis rates (Figure 8B), and upregulation of cleaved‐caspase‐3 and Bax with decreased Bcl‐2 (Figure 8C). BZD‐containing serum significantly reversed these changes. However, LY294002 attenuated these protective effects, resulting in increased apoptosis. Consistently, CORT reduced phosphorylation of PI3K, AKT, and mTOR, while BZD restored their phosphorylation; this effect was reversed by LY294002 (Figure 8D). These results indicate that BZD‐containing serum inhibits CORT‐induced neuronal apoptosis via activation of the PI3K/AKT/mTOR pathway. The corresponding full‐length western blot images are provided in Figure S2.

**FIGURE 8:**
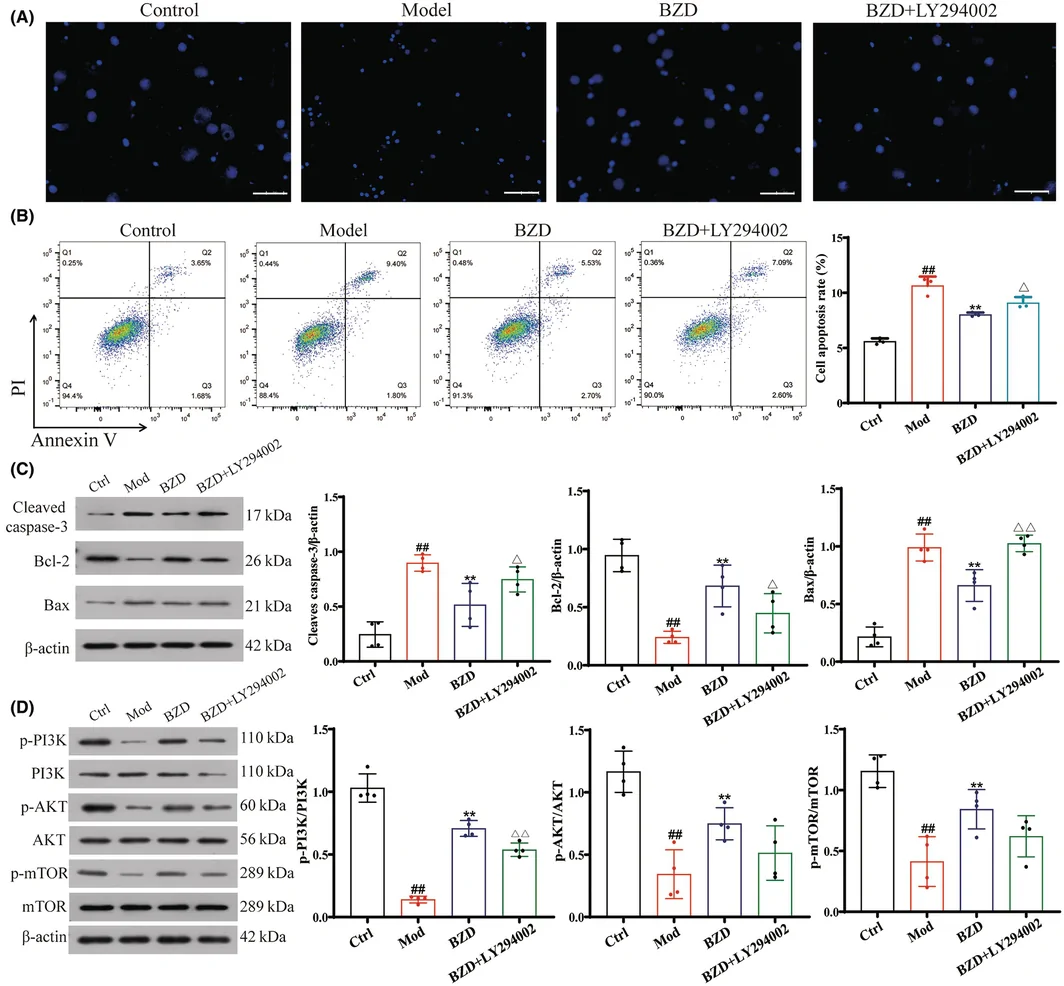
BZD‐containing serum protects hippocampal neurons against apoptosis through activation of the PI3K/AKT/mTOR pathway. (A) Hoechst 33342 staining (×200), scale bar: 100 μm. (B) Quantification of apoptosis by Annexin V‐FITC/PI flow cytometry (*n* = 3). (C) Representative Western blot bands and quantification of cleaved caspase‐3, Bax, and Bcl‐2 (*n* = 4). (D) Representative western blot and quantification of PI3K, AKT, mTOR and their phosphorylated forms (*n* = 4). Data are presented as mean ± SD. ^##^ *p* < 0.01 *vs*. control group; **p < 0.01 *vs*. model group; ^△^ *p* < 0.05, ^△△^ *p* < 0.01 *vs*. BZD group.

## 4 DISCUSSION

The present study demonstrates that BZD is safe and effective, exerting antidepressant effects and attenuating neuronal injury in an anxious‐depression mouse model induced by chronic restraint stress (CRS) combined with corticosterone (CORT) administration. Network pharmacology predicted the PI3K/AKT/mTOR pathway as a key mechanism underlying its therapeutic effects, which was further confirmed by in vivo and in vitro experiments. Inhibition by LY294002 indicated that these effects are closely associated with PI3K/AKT/mTOR‐mediated suppression of neuronal apoptosis.

A total of 41 chemical constituents were identified in BZD, mainly phenylpropanoids, flavonoids, and saponins, with saponins being predominant. Previous studies have shown that timosaponin BII and lily saponins exert anxiolytic and antidepressant‐like effects by modulating monoaminergic neurotransmission and exhibiting anti‐inflammatory and neuroprotective properties.^7^ Phenylpropanoids and flavonoids also possess anti‐inflammatory, antioxidant, and neuromodulatory activities, supporting the pharmacological basis of BZD.^29^

Anxious depression involves comorbid anxiety and depression and is influenced by multiple biological and environmental factors.^30^ Owing to its complexity, single‐factor models often fail to mimic its complexity. Therefore, CRS (6 h/day) combined with CORT (30 mg/kg) for 28 days was used to establish the model.^18^, ^31^, ^32^ This combined strategy is designed to integrate both sustained psychological stress and prolonged glucocorticoid exposure, thereby more closely mimicking the dysregulation of the hypothalamic–pituitary–adrenal (HPA) axis and the associated affective disturbances observed in anxious depression. Specifically, CRS induces persistent psychological stress and is widely used to evoke anxiety‐like behavioral alterations, whereas repeated CORT exposure models chronic hyperactivation of the HPA axis and glucocorticoid‐related depressive‐like states. The convergence of these two stressors has been reported to produce more robust and stable anxiety‐ and depression‐like phenotypes than either single‐factor approach alone. Behavioral results confirmed successful induction of anxious‐depressive phenotypes, which were significantly ameliorated by BZD.

Behavioral tests were conducted from less stressful to more stressful paradigms (OFT, EPM, SPT, FST), as recommended in previous studies.^19^ This testing strategy was adopted to minimize carryover effects and cumulative stress exposure, both of which may substantially influence behavioral outcomes. Repeated exposure to stressful behavioral procedures can activate the HPA axis, alter monoaminergic neurotransmission, and induce adaptive behavioral changes, thereby potentially confounding the interpretation of anxiety‐ and depression‐related behavioral measures. Therefore, arranging the tests in this order may have helped minimize potential confounding effects when interpreting the behavioral outcomes.

Restoration of hippocampal neuronal integrity is critical for treating anxious depression.^33^ As a central hub in emotional regulation, hippocampal dysfunction—manifesting as reduced neuronal density, diminished neuronal activity, and impaired synaptic plasticity—is considered a key neurobiological basis of anxiety and depressive behaviors.^34^ Effective interventions generally operate at two levels: first, reducing neuronal apoptosis by modulating Bcl‐2 family proteins or the caspase cascade^35^; and second, preserving synaptic structure to maintain functional neuronal connectivity.^36^ In this study, H&E, Nissl, and TEM analyses showed that BZD alleviated neuronal injury and improved synaptic structure in the CA1 region. BZD also downregulated Bax and cleaved caspase‐3 while upregulating Bcl‐2, supporting its anti‐apoptotic effects.

BZD originates from Jin Gui Yao Lue. Baihe nourishes yin and calms the spirit, whereas Zhimu clears heat and relieves irritability. Classical and modern TCM literature describes its efficacy in treating restlessness, insomnia, emotional dysregulation, and yin deficiency with internal heat—patterns that correspond closely to the TCM conceptualization of anxious depression.^6^ Pharmacological studies further indicate that lily extracts exert anxiolytic and antidepressant‐like effects through modulating neurotransmitters and improving oxidative stress and inflammatory status.^37^ Zhimu and its major saponins, such as timosaponin BII and BIII, also display marked antidepressant‐like activity in multiple behavioral paradigms and ameliorate neuroinflammation and monoaminergic dysfunction.^38^, ^39^ BZD has been shown to improve depressive behaviors in CUMS mice,^9^ modulate HPA axis function, and restore neuroinflammation and oxidative stress.^7^ These findings collectively support the antidepressant potential of BZD. Consistent with this, mangiferin and timosaponins identified in this study have been reported to exert neuroprotective and antidepressant‐related effects. Mangiferin has been shown to possess anti‐inflammatory and antioxidant properties and has been suggested to improve depression‐related behaviors, potentially through modulation of neuroinflammatory and oxidative stress processes.^40^ Timosaponin AIII, a representative steroidal saponin from *Anemarrhena asphodeloides*, has been reported to ameliorate learning and memory deficits in mice, indicating potential neuroprotective and cognition‐related benefits.^41^ Collectively, these findings support the potential contribution of these constituents to the neurobiological effects of BZD in the context of anxious depression. However, previous studies have mainly focused on component identification or target prediction, and lack systematic validation. By integrating chemical profiling, network pharmacology, and experimental verification, this study provides evidence for the mechanism of BZD in anxious depression.

Network pharmacology, grounded in systems biology, captures the multi‐component and multi‐target nature of TCM formulas by constructing comprehensive “herb‐compound‐target” networks.^42^ Using multiple databases and analytic tools, this study systematically identified key active constituents and potential targets of BZD relevant to anxious depression, ultimately highlighting the PI3K/AKT/mTOR pathway as a central node. Importantly, pathway prioritization in this study was not based solely on enrichment ranking; rather, it integrated statistical enrichment results with biological relevance and network topology characteristics. Although the PI3K/AKT/mTOR pathway was not necessarily the top‐ranked pathway in terms of enrichment score, it still met statistical significance criteria in KEGG analysis and included multiple mapped target genes identified in the network pharmacology analysis. In particular, key hub genes such as AKT and MTOR were directly involved in this signaling cascade, suggesting a central regulatory position within the constructed compound‐target network. Importantly, the relevance of this pathway is further supported by its close association with the pathophysiology of anxious depression. The PI3K/AKT/mTOR cascade is a canonical intracellular signaling pathway: PI3K activation generates PIP_3_ to recruit and activate AKT, which subsequently activates mTOR (including mTORC1 and mTORC2), regulating essential cellular processes such as growth, survival, metabolism, autophagy, apoptosis, and protein synthesis.^43^ In the nervous system, this pathway is critically involved in neuronal survival and metabolism,^44^ synaptic plasticity,^45^ long‐term potentiation (LTP),^46^ and neuronal morphology.^47^ Dysregulation of this pathway may impair brain function, neural plasticity, and emotion‐related circuits, thereby contributing to psychiatric symptoms such as depression and anxiety^48^, ^49^ Several primary constituents of BZD are known to modulate this pathway: mangiferin regulates PI3K/AKT/mTOR activity to influence proliferation, apoptosis, and autophagy,^50^ and regaloside A upregulates BDNF–TrkB signaling accompanied by enhanced phosphorylation of PI3K and AKT, suggesting that it may exert neuroprotective or antidepressant‐like effects via the BDNF–TrkB→PI3K/AKT axis.^51^

Overall, this study demonstrates that activation of the PI3K/AKT/mTOR pathway is critical for BZD to inhibit hippocampal neuronal apoptosis and improve anxious‐depressive behaviors. However, several limitations remain. Only the chemical constituents were characterized, without identification of bioactive or brain‐penetrant components, and the blood–brain barrier permeability, brain distribution, and tissue concentrations of BZD constituents were not directly determined. In addition, the in vivo and in vitro experiments were performed using different rodent species, with C57BL/6 mice used for the animal studies^16^ and primary hippocampal neurons isolated from SD rats for the cell experiments. Although primary rat hippocampal neurons are widely used for mechanistic studies,^25^ the use of species‐consistent models in future studies would further strengthen the robustness and translational relevance of our findings. Furthermore, only male mice were used to minimize variability associated with estrous cycle‐related hormonal fluctuations^52^, ^53^, ^54^; however, given the higher prevalence of anxiety and depression in females, potential sex‐specific responses to BZD cannot be excluded, which may limit the generalizability of the findings.^55^ Future studies will focus on identifying the absorbed bioactive constituents and brain‐penetrant components of BZD, together with their pharmacokinetic properties, brain distribution, and tissue exposure levels. Such investigations will help clarify the material basis and central mechanisms underlying the therapeutic effects of BZD and further support its pharmacological and translational application.

## 5 CONCLUSION

This study integrates UPLC‐Q‐TOF‐MS/MS, network pharmacology, and multi‐level experimental validation to demonstrate that BZD ameliorates the anxiety‐ and depression‐like behaviors induced by CRS combined with CORT by activating the PI3K/AKT/mTOR pathway and inhibiting hippocampal neuronal apoptosis. These findings clarify the molecular mechanism underlying this classical formula and provide experimental evidence and potential targets for its clinical application in mood disorders.

## AUTHOR CONTRIBUTIONS

**Yuanshan Han:** Conceptualization; methodology; investigation; formal analysis; writing – original draft. **Kexin He:** Investigation; data curation; formal analysis; visualization; writing – original draft; validation. **Haolan Yang:** Methodology; investigation; data curation; software. **Jiacheng Dai:** Investigation; data curation; formal analysis; writing – original draft; validation. **Ronglin Chen:** Visualization; data curation. **Yang Liu:** Investigation; data curation. **Yuhong Wang:** Resources; data curation. **Lin Tang:** Supervision; project administration; writing – review and editing; funding acquisition. **Hongqing Zhao:** Conceptualization; methodology; supervision; project administration; funding acquisition; writing – review and editing.

## FUNDING INFORMATION

Funding for this work came from the National Natural Science Foundation of China (Nos. 82675753, 82575029, 82505002, and 82104836), Innovation Research Group Project of Hunan Natural Science Foundation (No. 2024JJ1007), Natural Science Foundation of Hunan Province (No. 2026JJ50265), Key Project of the Education Department of Hunan Province (No. 25A0282), Open Fund of National Key Laboratory Cultivation Base of Chinese Medicinal Powder & Innovative Medicinal Jointly Established by Province and Ministry (No. 25PTKF1002) and Science and Technology Talent Promotion Project of Hunan Province (No. 2023TJ‐N22).

## CONFLICT OF INTEREST STATEMENT

The authors declare that the research was conducted in the absence of any commercial or financial relationships that could be construed as a potential conflict of interest.

## ETHICS STATEMENT

Approval for all animal‐related procedures was granted by the Animal Ethics Committee of Hunan University of Chinese Medicine (Approval no. LLBH‐202308120003).

## PERMISSION TO REPRODUCE MATERIAL FROM OTHER SOURCES

No material from other sources was reproduced in this manuscript; therefore, no permission is required.

## Supporting information


**Supplementary Material 1.** Primer sequences, antibodies used in western blot, and mass spectra of BZD compounds.
**Supplementary Table 1**. Primer nucleotide sequence list.
**Supplementary Table 2**. Dilutions were optimized for cell and tissue lysates separately. Abbreviations: CST, Cell Signaling Technology; Affinity, Affinity Biosciences; Proteintech, Proteintech Group, Inc.
**Supplementary Table 3**. Mass spectrum information of compounds from BZD. Compounds marked with * correspond to those indicated in Figure 1C of the main text.

**Supplementary Material 2.** Uncropped image of western blot membranes.
Figure S1. Original unprocessed images of WB for Figure 6.
Figure S2. Original unprocessed images of WB for Figure 8.

## Data Availability

The datasets used and analyzed during the current study are available from the corresponding author on reasonable request.
