# Supplementary material for: Baihe Zhimu Decoction ameliorates anxious depression via PI3K/AKT/mTOR pathway‐mediated neuroprotection and inhibition of hippocampal neuronal apoptosis
Source: Animal Model Exp Med. 2026 Oct 12:10.1002/ame2.70295. Online ahead of print. doi: 10.1002/ame2.70295 (PMC13655827; doi:10.1002/ame2.70295)
Supplement: Supplementary file 1 [file AME2-9999-0-s001.pdf]

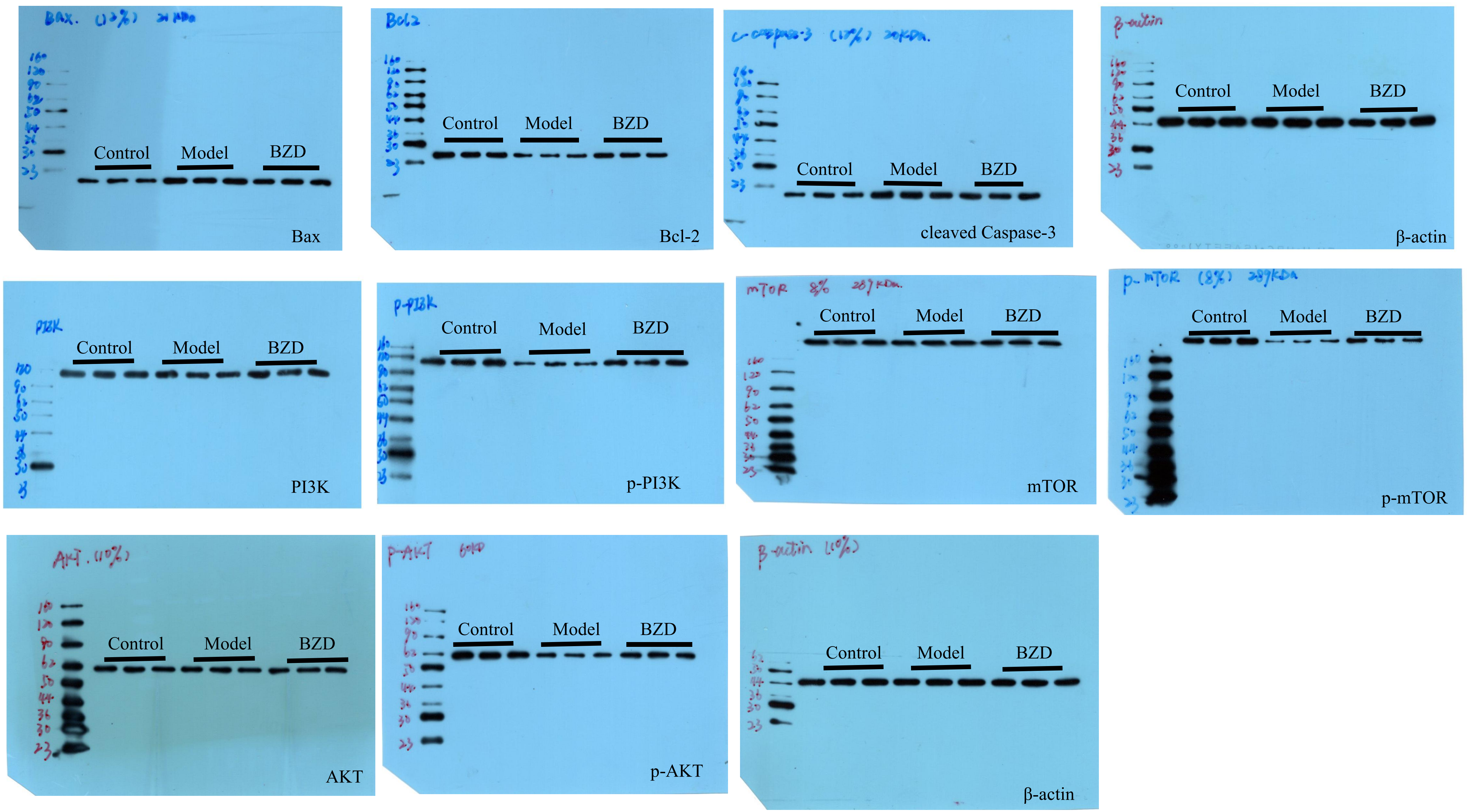

Fig.S1. Original unprocessed images of WB for Fig. 6.

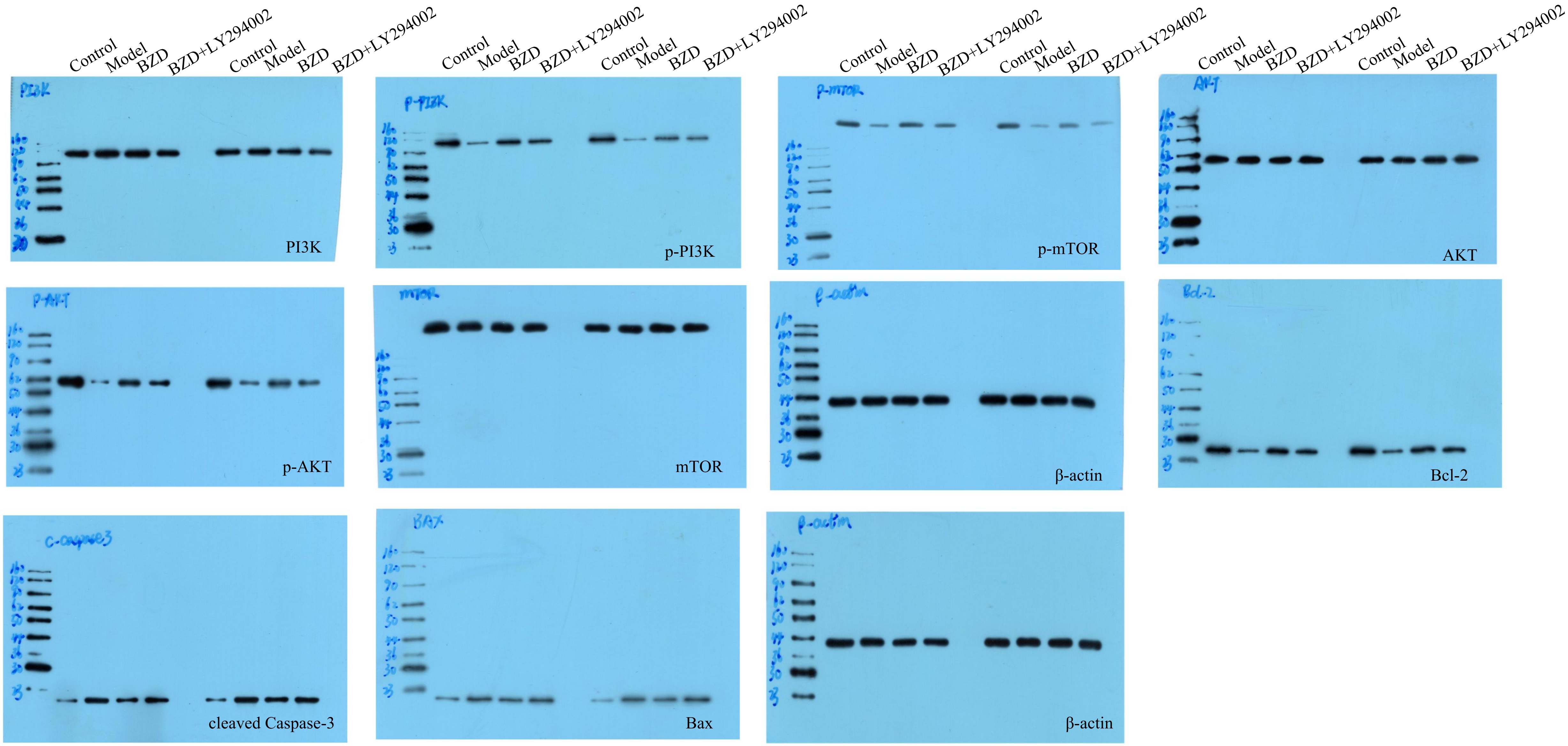

Fig.S2. Original unprocessed images of WB for Fig. 8.

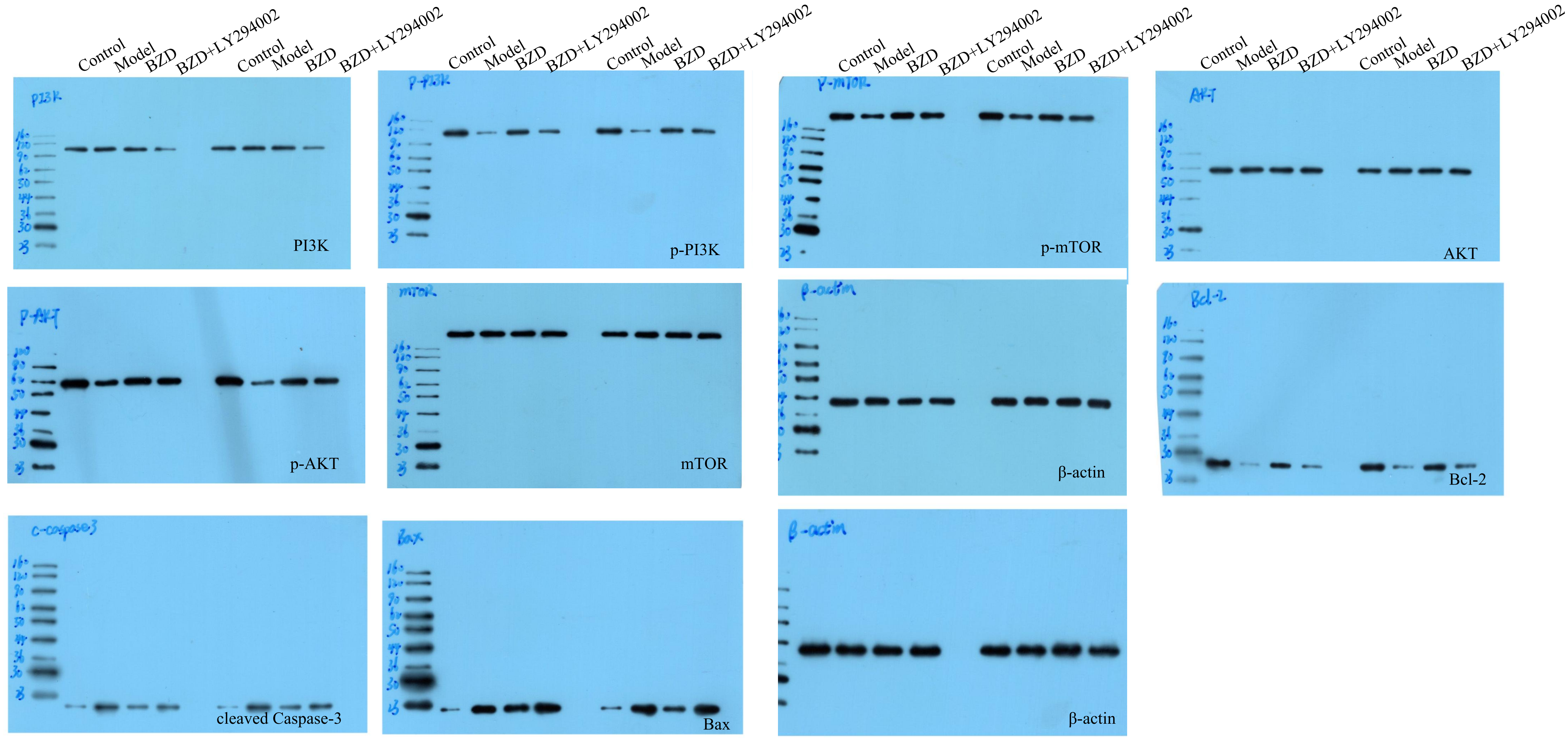

Fig.S2. Original unprocessed images of WB for Fig. 8.
