# Supplementary material for: Baihe Zhimu Decoction ameliorates anxious depression via PI3K/AKT/mTOR pathway‐mediated neuroprotection and inhibition of hippocampal neuronal apoptosis
Source: Animal Model Exp Med. 2026 Oct 12:10.1002/ame2.70295. Online ahead of print. doi: 10.1002/ame2.70295 (PMC13655827; doi:10.1002/ame2.70295)
Supplement: Supplementary file 2 [file AME2-9999-0-s002.doc]

**Supplementary Table 1.** Primer nucleotide sequence list.

| Primers | Forward sequence (5’→3’) | Reverse sequence (5’→3) |
| --- | --- | --- |
| AKT1 | F: AAG CCT TCA TGC AGG AGC A | TCC ATG CTT CTT GGC GTC |
| mTOR | F: GCA AGC GCA ATC TCT GGT | GCG CAA CAC TGA AAA CCT C |
| Bax | F: CCT CAG GCA TCT TTT GCT T | CTT GTG GTA GGT GTC GAA GG |
| Bcl2 | F: CGA CAG GAT CGC TGC CTT | GCA TGC AAA TTC CTT TCC AG |
| Caspase-3 | F: AAT AAC CAC GCT ACT GGT GC | TCT CTG TCC CTC CAT TGT GA |
| β-actin | F: TCC ACC ACC CAG ACA TCA TG | TCA CCA GGC GTC CTT CCT A |

**Supplementary Table 2.Dilutions were optimized for cell and tissue lysates separately. Abbreviations: CST, Cell Signaling Technology; Affinity, Affinity Biosciences; Proteintech, Proteintech Group, Inc.**

| Target | Sample | Dilution | Cat. No. | Source |
| --- | --- | --- | --- | --- |
| p-PI3K | Cell/Tissue | 1:2000 | AF4369 | Affinity |
| PI3K | Cell/Tissue | 1:1000 | 20584-1-AP | Proteintech |
| p-AKT | Cell | 1:10000 | 66444-1-Ig | Proteintech |
|  | Tissue | 1:2000 | 4060 | CST |
| p-mTOR | Cell | 1:10000 | 67778-1-Ig | Proteintech |
|  | Tissue | 1:1000 | Ap0094 | ABclonal |
| AKT | Cell | 1:10000 | 10176-2-AP | Proteintech |
|  | Tissue | 1:1000 | 4691 | CST |
| mTOR | Cell/Tissue | 1:10000 | 66888-1-Ig | Proteintech |
| Bax | Cell | 1:1000 | 50599-2-Ig | Proteintech |
|  | Tissue | 1:2000 | cy5059 | Abways |
| Bcl-2 | Cell/Tissue | 1:3000/4000 | 26593-1-AP | Proteintech |
| C-Caspase-3 | Cell/Tissue | 1:2000 | AF7022 | Affinity |
| β-actin | Cell/Tissue | 1:15000/20000 | GB11001 | Servicebio |

**Supplementary Table 3.** Mass spectrum information of compounds from BZD.Compounds marked with * correspond to those indicated in Figure 1C of the main text.

| **No.** | **English Name** | **tR/min** | **Ions**  **Mode** | **Molecular Formula** | **Molecular Weight** | **Error** | **Fragment Ions** | **Attribution** |
| --- | --- | --- | --- | --- | --- | --- | --- | --- |
| 1 | Sucrose | 1.069 | +/- | C_12_H_22_O_11_ | 342.1162 | 1 | +:284,266,248,162,138 | *Lilium lancifolium* Thunb. |
|  |  |  |  |  |  |  | -:297,209,179,135 |  |
| 2 | Glomeratose A | 1.203 | + | C_18_H_24_O_10_ | 400.1369 | -0.83 | +:418,386,356,256,162,146 | *Lilium lancifolium* Thunb. |
| 3 | Rhodiocyanoside A | 1.81 | - | C_11_H_17_NO_6_ | 259.1056 | -1.72 | -:286,274,245, | *Lilium lancifolium* Thunb. |
| 4 | Phenylalanine | 3.069 | +/- | C_9_H_11_NO_2_ | 165.079 | 1.34 | +:121,104, | *Lilium lancifolium* Thunb. |
|  |  |  |  |  |  |  | -:137,119,100 |  |
| 5 | Sitosterol | 5.876 | +/- | C_10_H_19_NO_5_ | 233.1263 | 0.94 | +：216,198,180.162,112,104,86 | *Lilium lancifolium* Thunb. |
|  |  |  |  |  |  |  | -:214,188,146,102,84 |  |
| 6* | Neomangiferin | 8.067 | +/- | C_25_H_28_O_16_ | 584.1377 | 0.85 | +:567,489,435,369,327,303,273 | *Anemarrhena asphodeloides* Bunge |
|  |  |  |  |  |  |  | -:523,493,463,403,373,331,301 |  |
| 7 | N,N'-Diacetyltyramine | 9.347 | - | C_20_H_20_N_2_O_8_ | 416.122 | -0.38 | -:235,179,161,135,113,101 | *Lilium lancifolium* Thunb. |
| 8 | 2-Hydroxypropylsucrose | 9.827 | +/- | C_15_H_28_O_12_ | 400.1581 | 2.16 | +:352,289,221,187,147 | *Lilium lancifolium* Thunb. |
|  |  |  |  |  |  |  | -:327,219,163,145,119,101 |  |
| 9* | Mangiferin | 10.672 | +/- | C_19_H_18_O_11_ | 422.0849 | 0.59 | +:405,387,357,327,285,273 | *Anemarrhena asphodeloides* Bunge |
|  |  |  |  |  |  |  | -:403,385.,355,331,301,275,259 |  |
| 10* | Isomangiferin | 10.772 | +/- | C_19_H_18_O_11_ | 422.0849 | 3.68 | +:405,387,357,327,285,273 | *Anemarrhena asphodeloides* Bunge |
|  |  |  |  |  |  |  | -:403,385.,355,331,301,275,259 |  |
| 11 | Regaloside A | 11.599 | +/- | C_18_H_24_O_10_ | 400.1369 | 0.05 | +:393,376,306,147 | *Lilium lancifolium* Thunb. |
|  |  |  |  |  |  |  | -:331,239,163,145 |  |
| 12 | Regaloside D | 11.782 | - | C_18_H_24_O_10_ | 400.1369 | 3.68 | -:335,253,219,163,145 | *Anemarrhena asphodeloides* Bunge |
| 13 | Regaloside F | 12.577 | - | C_19_H_26_O_11_ | 430.1475 | -0.8 | -:401,357,323,276,193 | *Lilium lancifolium* Thunb. |
| 14 | p-Coumaric Acid | 13.239 | - | C_9_H_8_O_3_ | 164.0473 | -3.63 | -:136.9243,119.0366 | *Lilium lancifolium* Thunb. |
| 15 | Myricetin 3-O-Galactoside | 13.786 | - | C_21_H_20_O_13_ | 480.0904 | 3.63 | -:461,417,389,359,345,315,287 | *Lilium lancifolium* Thunb. |
| 16 | Regaloside E | 14.051 | - | C_20_H_26_O_12_ | 458.1424 | 0.33 | -:415, 397, 319, 179 ,161 | *Lilium lancifolium* Thunb. |
| 17 | Alfaloside | 14.597 | +/- | C_21_H_20_O_10_ | 432.1056 | -0.28 | +:415,397,367,337,313,283,165 | *Anemarrhena asphodeloides* Bunge |
|  |  |  |  |  |  |  | -:353,341,311,283,239 |  |
| 18 | Quercetin 3-O-Glucoside | 14.697 | +/- | C_21_H_20_O_10_ | 432.1056 | -0.31 | +:415,397,379,337,313,283 | *Anemarrhena asphodeloides* Bunge |
|  |  |  |  |  |  |  | -:413,383,353,341,311,283,239 |  |
| 19 | Diosgenin | 15.422 | + | C33H52O8 | 576.3662 | -0.5 | +:546,502,477, 458,330 | *Anemarrhena asphodeloides* Bunge |
| 20* | Timosaponin B | 15.74 | +/- | C_20_H_26_O_11_ | 442.1475 | -0.23 | +：401, 383, 357, 301,147 | *Lilium lancifolium* Thunb. |
|  |  |  |  |  |  |  | -:399,381,303,163,145. |  |
| 21 | Regaloside B | 16.105 | +/- | C_20_H_26_O_11_ | 442.1475 | 0.02 | +：401, 383, 357, 301,147 | *Lilium lancifolium* Thunb. |
|  |  |  |  |  |  |  | -:399,381,303,163,145. |  |
| 22 | Timosaponin E1 | 16.383 | +/- | C_45_H_76_ O_20_ | 936.493 | 1.45 | +:757,595.,433,289 | *Lilium lancifolium* Thunb. |
|  |  |  |  |  |  |  | -:891,773.,611,449 |  |
| 23 | Geranyl formate | 16.942 | + | C11H18O2 | 182.137 | 0.45 | +:155,127 | *Lilium lancifolium* Thunb. |
| 24 | Timosaponin E | 17.121 | +/- | C_45_H_76_ O_20_ | 936.493 | 0.67 | +:919,757,595, 433,271 | *Anemarrhena asphodeloides* Bunge |
|  |  |  |  |  |  |  | -:733,611,449,263,179 |  |
| 25 | Timosaponin D | 17.792 | +/- | C_45_H_74_O_19_ | 918.4824 | -0.01 | +:879,815,758.,596 | *Anemarrhena asphodeloides* Bunge |
| 26* | Timosaponin II | 18.105 | +/- | C_45_H_76_O_19_ | 920.4981 | 0.46 | +:903,741,579,417,273 | *Anemarrhena asphodeloides* Bunge |
|  |  |  |  |  |  |  | -:757,595,433,263 |  |
| 27* | Timosaponin BIII | 19.322 | +/- | C_45_H_74_O_18_ | 902.4825 | 1.82 | +:741,579,417,381,273,155,145 | *Lilium lancifolium* Thunb. |
|  |  |  |  |  |  |  | -:755,595,247,163,89 |  |
| 28 | Timosaponin D | 20.18 | - | C_45_H_74_O_19_ | 918.4824 | -1.71 | +:879,815,758.,596 | *Anemarrhena asphodeloides* Bunge |
| 29 | 17-hydroxygracillin | 20.594 | +/- | C_45_H_72_O_18_ | 900.4719 | 0.84 | -:739,577,415,273,99 | *Lilium lancifolium* Thunb. |
|  |  |  |  |  |  |  | -:737,575,293,161 |  |
| 30 | Timosaponin C | 21.123 | +/- | C_45_H_74_O_18_ | 902.4875 | -1.14 | +：741,579,417,373,145 | *Anemarrhena asphodeloides* Bunge |
|  |  |  |  |  |  |  | -:739,577,323,221,101 |  |
| 31 | Timosaponin H2 | 21.257 | +/- | C_45_H_74_O_18_ | 902.4875 | -2.56 | +:874,860,741,579,417,255 | *Anemarrhena asphodeloides* Bunge |
|  |  |  |  |  |  |  | -:739,673,577,323,221 |  |
| 32 | Smilaxin C | 22.664 | +/- | C_44_H_72_O_17_ | 872.477 | 0.29 | +:711,579,457,417,273 | *Lilium lancifolium* Thunb. |
|  |  |  |  |  |  |  | -:709,547,399,293,101 |  |
| 33 | Pinolenic Acid | 22.946 | - | C_18_H_34_O_5_ | 330.2406 | -0.4 | -:312,270,253 | *Anemarrhena asphodeloides* Bunge |
| 34 | Timosaponin A Ⅳ | 23.537 | +/- | C_39_H_64_O_13_ | 740.4347 | -0.63 | +：741,417,381,363,271,253,145 | *Anemarrhena asphodeloides* Bunge |
|  |  |  |  |  |  |  | -：595,529,433,161 |  |
| 35 | Ophiogenin-3-O-α-L-rhamnosyl-(1→2)-β-D-glucoside | 23.834 | +/- | C_39_ H_62_O_14_ | 754.414 | -0.36 | +：737,701,539,447,309,237 | *Lilium lancifolium* Thunb. |
|  |  |  |  |  |  |  | -:607,307,247,205,163 |  |
| 36 | Timosaponin A1 | 24.512 | + | C_33_H_54_O_8_ | 578.3819 | 1.45 | +:543,459,417,318,273,167,115 | *Anemarrhena asphodeloides* Bunge |
| 37 | Timosaponin III | 24.655 | +/- | C_39_ H_64_ O_14_ | 756.4296 | 0.14 | +:629,595,433,397,293,283,253 | *Anemarrhena asphodeloides* Bunge |
|  |  |  |  |  |  |  | -:627,593,527,473,323,161.101 |  |
| 38 | Aspafilioside A | 24.856 | +/- | C_38_ H_62_ O_12_ | 710.4241 | -0.22 | +:595,433,397,293, 253 | *Anemarrhena asphodeloides* Bunge |
|  |  |  |  |  |  |  | -:593,527,323,161,113,101 |  |
| 39 | Timosaponin III | 25.929 | +/- | C_39_ H_64_ O_14_ | 756.4296 | 0.14 | +:629,595,433,397, 283,253 | *Anemarrhena asphodeloides* Bunge |
|  |  |  |  |  |  |  | -:627,593,527,473,323,161.101 |  |
| 40 | Timosaponin | 27.807 | +/- | C_40_H_68_O_15_ | 788.4558 | 1.21 | +:579,417,399,279,221 | *Anemarrhena asphodeloides* Bunge |
|  |  |  |  |  |  |  | -:577,323,161,101 |  |
| 41* | Timosaponin A-III | 28.813 | +/- | C_39_H_64_O_13_ | 740.4347 | -0.12 | +: 579,417,381,325,273,255,145 | *Anemarrhena asphodeloides* Bunge |
|  |  |  |  |  |  |  | -: 577,511,457,323,161,143,101 |  |
